# Bile constituents in hibernating golden-mantled ground squirrels (*Spermophilus lateralis*)

**DOI:** 10.1186/1476-5926-8-2

**Published:** 2009-05-26

**Authors:** Julie A Baker, Frank van Breukelen

**Affiliations:** 1 School of Life Sciences, University of Nevada Las Vegas, 4505 Maryland Parkway, Las Vegas Nevada 89154 USA

## Abstract

**Background:**

Golden-mantled ground squirrels (*S. lateralis*) are anorexic during the winter and survive by exploiting hibernation to reduce energetic demands. The liver normally plays a critical role in fueling and regulating metabolism and one might expect significant changes in hepatobiliary function with hibernation. We analyzed bile collected from animals in summer, animals in winter that were either torpid, active between bouts of torpor, or which failed to enter hibernation in order to characterize the effects of hibernation on hepatobiliary function *per se*.

**Results:**

Surprisingly, hibernator bile did not differ from summer squirrel bile in key characteristics including [bile acids], [cholesterol], [free fatty acids], [lecithin], and osmolality. One major distinction between summer and winter squirrels was that winter squirrels experience >5 fold increases in [bilirubin]. Such an increase may have significant physiological consequences that could aid in survivorship of torpor. Animals that failed to hibernate, despite being anorexic, were very similar to summer squirrels in all measured parameters except they had lower bile acid and lecithin concentrations.

**Conclusion:**

The data indicate that despite extended anorexia, differences in metabolic fuel privation, and bouts of reduced body temperatures, hibernators normally do not experience broad changes in hepatobiliary function.

## Background

Hibernation is a strategy employed by many different mammals presumably as a means for energy conservation during periods of great thermal stress and limited food resources [1,2]. Ground squirrels of the genus *Spermophilus *are exemplary hibernators. Their winter seasons are characterized by bouts of torpor wherein body temperature may approach ambient to as low as -2°C [3,4] and metabolic rates may be as low as 1% of the active rate [5]. These torpor bouts may last 1–3 weeks and are interrupted by brief (~20–24 h) sojourns to body temperatures and metabolic rates typical of an active animal.

During the winter, golden-mantled ground squirrels *(S. lateralis*) are anorexic. Even when housed with free access to food, very few of these animals will eat for the entire ~6 month hibernation season (personal observations). Instead, animals rely on immense fat stores that were gained in an anticipatory period during late summer [2]. Hibernating animals utilize a primarily fat-based metabolism as reflected by a typical respiratory quotient (RQ) of 0.71 but employ a more carbohydrate-based metabolism (RQ = ~0.9) during the interbout-arousal [6]. As expected, the consequences of the anorexic period include a profound disuse atrophy of the gut and the physiology of this atrophy has been well described [7-9]. Although a large number of studies have used the liver as a model organ for examining the effects of hibernation on various metabolic activities such as protein synthesis, we are unaware of any studies that have examined digestive hepatobiliary function *per se *during hibernation. One might expect dramatic changes in liver function due to the extended anorexia, metabolic fuel privation, and severe physiological conditions inherent to hibernation.

Mortality rates during hibernation are high; as many as 40–70% of squirrels fail to emerge from the burrow the following spring [10]. Although no data are available as to the cause of this mortality, likely explanations include a metabolic disorder or a lack of energetic supplies to withstand the extended anorexia. In maintaining laboratory colonies for other experiments, we occasionally encounter some animals that fail to hibernate (< 5% of all animals; personal observations). These animals typically lose weight quickly and die during the winter. We observed a marked difference in bile color of these animals. As a result of this observation and to characterize hepatic function during hibernation, we examined the constituents of bile in active and hibernating squirrels.

## Results

**Figure 1:**
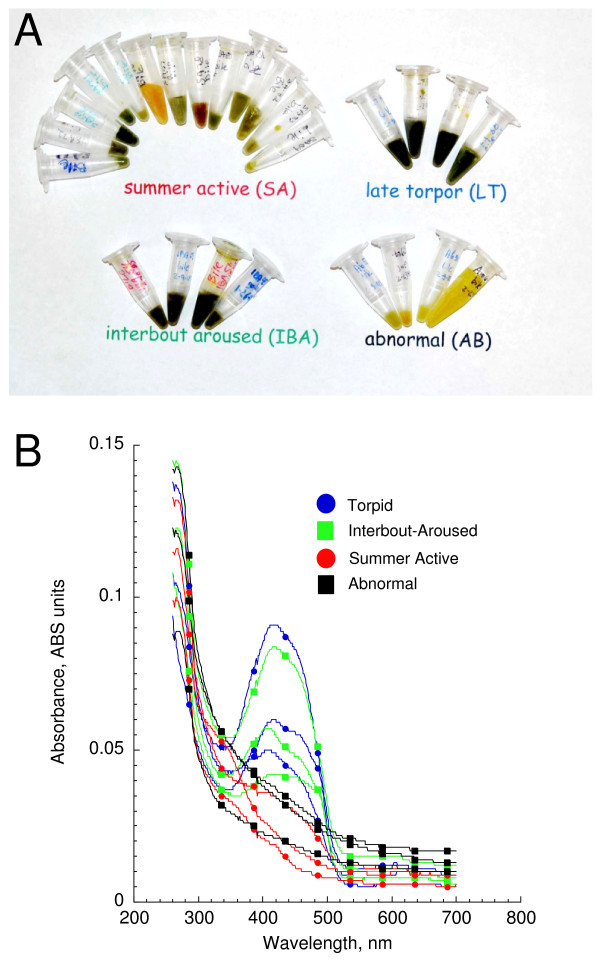
**Gallbladder bile color varies by season and hibernation**. A) Photograph of bile collected from golden-mantled ground squirrels *(Spermophilus lateralis*) as a function of state. Bile was collected from squirrels collected monthly (2–3 squirrels per month) from May (left) until September (right; summer active, SA), squirrels during winter that were torpid (T) when body temperature was ~5°C, squirrels during the euthermic period between bouts of torpor (interbout-aroused; IBA), and squirrels that were sampled in winter but had failed to hibernate (abnormal, AB). As an indication of approximate volumes, microcentrifuge tubes contain all of the collected bile for each animal except one AB animal (full tube on lower right; ~2.5 ml of bile was collected from that animal). B) Spectral characteristics of bile as a function of state. Each line represents one animal. Data are depicted for 3 animals of each state and only every 50^th ^symbol is plotted for clarity.

In our experience, golden-mantled ground squirrels have proven to be very reliable hibernators in the laboratory. Indeed, we have observed hibernation at ambient temperatures of 20°C, with a variety of lighting conditions, and in the presence of free access to food. Rarely, some animals fail to hibernate (< 5% of all animals; personal observations). Despite access to food, these animals are generally anorexic and quickly lose weight and die during the winter. In the course of our other recent studies, we found 4 such animals. Following sacrifice and subsequent tissue collection, we noted a seemingly abnormal appearance to their bile (Fig. 1A). While other winter squirrels (torpid – T and interbout-aroused – IBA) consistently had deep green bile, the squirrels that failed to hibernate (deemed abnormal – AB) had bright yellow, almost fluorescent bile despite having been sampled at the same time of year. These squirrels had little to any gut contents consistent with the anorexia normally associated with the hibernation season. As indicated in Fig. 1A, collected bile volumes were quite varied throughout the year but rarely exceeded 500 μl. However, approximately 2.5 ml of bile was collected for one AB animal (Fig. 1A- right). Summer active (SA) squirrels had more varied bile colors as might be expected given the effect of diet on bile color [11,12]. However, our sampling of squirrels from early spring to late summer revealed no simple association of bile color with a given time period (Fig. 1A). Spectral analyses revealed that bile from T and IBA animals contained a peak at approximately 350–500 nm that was not present in either SA or AB squirrels (Fig. 1B). Remarkably, despite having a seemingly fluorescent yellow outward appearance, AB bile was relatively inactive spectrophotometrically. The wide range of bile color in summer had little effect on spectral characteristics (data not shown).

**Figure 2:**
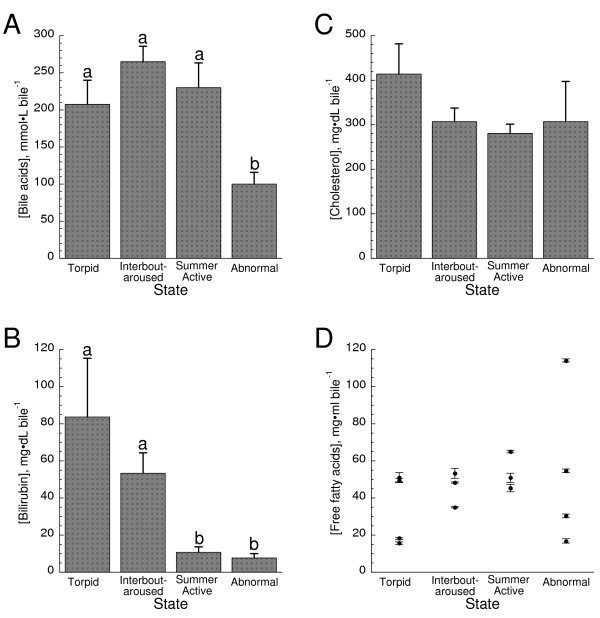
**Bile constituents as a function of hibernation state**. A) Bile acid concentrations in bile as a function of state. Values represent means ± SE from T (n = 3), IBA (n = 3), SA (n = 3), and AB squirrels (n = 4). AB was significantly lower than all other states (ANOVA, p < 0.05). When different, letters above error bars denote significant differences. B) Bilirubin concentration in bile as a function of state. Values represent means ± SE from T (n = 3), IBA (n = 3), SA (n = 5), and AB squirrels (n = 4). There were no significant differences between T and IBA or between SA and AB. All other values are significantly different (ANOVA, p < 0.05). C) Bile cholesterol concentration as a function of state. Values represent means ± SE from T (n = 3), IBA (n = 3), SA (n = 13), and AB squirrels (n = 4). There were no significant differences (ANOVA, p > 0.05). D) Free fatty acid concentrations in bile as a function of state. Values depicted are from each individual animal (means ± SE) to demonstrate individual variation and represent T (n = 3), IBA (n = 3), SA (n = 3), and AB squirrels (n = 4). There were no significant differences (ANOVA, p > 0.05).

Bile acids are produced in the liver by the oxidation of cholesterol and serve important roles in eliminating cholesterol from the body and the emulsification of lipids [13,14]. Under normal physiological conditions, most bile acids are reabsorbed from the ileum and therefore values typically represent the reabsorption kinetics of bile acids as a function of enterohepatic circulation. Bile acid concentrations were significantly lower in the AB animals and were less than 50% of values recorded for the other groups (Fig. 2A). Bilirubin is the product of erythrocyte and hemoglobin turnover [13]. Concentrations of bilirubin were much lower (at least 5-fold) in both SA and AB squirrels as compared to winter hibernators (Fig. 2B). However, there were no significant differences found for either cholesterol or free fatty acid concentrations as a function of state (Fig. 2C,D). It should be noted that there was marked individual variation in the AB group squirrels for biliary free fatty acids with one squirrel demonstrating about a two fold higher concentration (not the squirrel with the large volume of bile).

**Figure 3:**
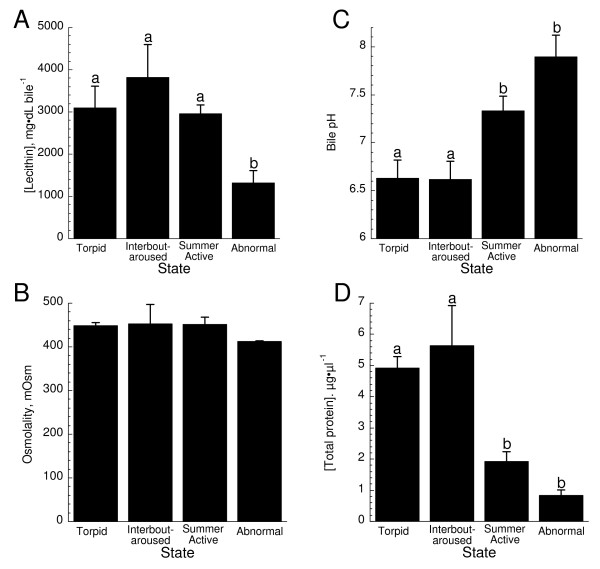
**Bile constituents as a function of hibernation state**. A) Bile lecithin/phosphatidylcholine concentration as a function of state. Values represent means ± SE from T (n = 3), IBA (n = 3), SA (n = 3), and AB squirrels (n = 4). AB was significantly lower than all other states (ANOVA, p < 0.05). When different, letters above error bars denote significant differences. B) Bile osmolality as a function of state. Values represent means ± SE from T (n = 6), IBA (n = 3), SA (n = 11), and AB squirrels (n = 4). There were no significant differences (ANOVA, p > 0.05). C) pH of bile measured at 37°C as a function of state. Values represent means ± SE from T (n = 4), IBA (n = 4), SA (n = 10), and AB squirrels (n = 4). All values are significantly different except between T and IBA (ANOVA, p > 0.05). D) Total protein concentration in bile as a function of state. Values represent means ± SE from T (n = 3), IBA (n = 3), SA (n = 5), and AB squirrels (n = 4). There were no significant differences between T and IBA or between SA and AB. All other values are significantly different (ANOVA, p < 0.05).

Lecithin or phosphatidylcholine was significantly lower in the AB group as compared to all other squirrels (Fig. 3A). A major function of lecithin is in the excretion of cholesterol during normal metabolism [13]. Osmolality was unchanged as a function of state (Fig. 3B). Torpor state had a significant effect on pH (Fig. 3C). Bile from winter hibernators (T and IBA) was significantly more acidic than either SA or AB bile. Indeed, hibernator bile had over 10 fold higher H^+ ^concentration than AB bile (> 1.2 pH units). Bile protein concentration was significantly different as a result of state: hibernators (T and IBA) had approximately 5 fold higher protein levels than their AB counterparts (Fig. 3D). AB animals were more similar to SA squirrels.

## Discussion

The winter season for a hibernator is marked by extended anorexia [2]. Given the liver's role in fueling metabolism, we hypothesized that there might be changes in liver function as a function of hibernation and associated anorexia. We present here the first data on the effects of hibernation on gallbladder bile constituents. Although there were no significant differences in bile constituents between torpid and aroused winter squirrels, a few differences were found between the winter squirrels and summer squirrels. Except for [bilirubin], these differences did not involve critical indicators of metabolic function. Finally, we examined bile in winter animals that failed to enter torpor and found that they had significantly lower [bile acids] and [lecithin] as compared to all other groups. We discuss below the implications of these findings.

To satisfy its energetic demands during winter, a hibernator relies on stored lipids. Changes in lipid pools have dramatic effects on the ability of a hibernator to successfully employ torpor; increased dietary poly-unsaturated fatty acids increase torpor bout usage, length, and depth [15]. A major function of the liver, and more specifically bile, is to facilitate digestion and absorption of lipids from the intestine. However, what happens to hepatobiliary function when there are no foodstuffs in the gut? The anorexia of hibernation allows for an examination of an extended (months long) anorexia not available with almost any other mammalian system except denning bears. In our study, we were surprised by the few changes in the bile constituents between summer and normal winter squirrels (both IBA and T); important indicators of metabolic function such as biliary [bile acids], [cholesterol], [free fatty acids], and [lecithin] were unchanged despite the months long anorexia experienced by winter squirrels (Figs. 2, 3). Although biliary changes as a function of season were found for [bilirubin], spectral characteristics, pH, and [total protein], the roles that most of these other factors have as indicators of hepatobiliary function seems less robust (Figs. 1, 2, 3). For instance, biliary pH is known to be quite variable [e.g., [16]]. However, the effect of altered pH is not well established; very low pH is associated with oxidative damage [17] and moderately high pH may be associated with gallstone formation [18]. In bears, significant increases in both biliary cholesterol and lecithin were noted as a function of season but it is unclear when captive or wild bears were used so dietary considerations may have biased the results [19]. We also note that bear denning is a markedly distinct physiological state from true mammalian hibernation, e.g., reductions of body temperatures in bears are modest (< 6°C) and most metabolic processes including kidney function are maintained [20].

Canalicular secretion of bile acids or other osmolytes generates an osmotic gradient for osmotic flow of water into bile [13,14]. As a result, bile flow is usually directly related to bile acid/salt secretion. Since high levels of bile acids would suggest high biliary flow rates, it is not surprising that [bile acids] were high in summer squirrels that were actively eating when sampled (Fig. 2A). What was puzzling was that bile acid concentrations were also high in winter hibernators (T and IBA) but not in those winter squirrels that failed to hibernate (AB; Fig. 2A). All three winter groups were anorexic. One might expect very little need for secretion of bile during an extended anorexic period and the decreased bile acids in AB animals may indeed reflect reduced bile production. However, the same argument should also apply to the hibernators unless there is a functional difference in hepatobiliary physiology between squirrels that hibernate and those that fail to hibernate. One such difference may be gallbladder contractility. Fasting normally results in sustained suppression of gallbladder contractility [21]. It follows that as a consequence of little to no gut activity, gallbladder contractility may be minimal in hibernators. If the contents of the gallbladder are not expelled, normal physiological function would result in a concentrating effect as water is removed from the gall bladder, e.g., gallbladder bile is oftentimes more than 20 fold more concentrated than hepatic bile [13]. A simple snapshot of bile constituents as provided here cannot address if there is enterohepatic circulation of bile acids during the winter season. Of note is that while bilirubin concentrations were high in winter hibernators, they were low in both SA and AB animals (Fig 2B) further suggesting gallbladder contractions in these animals but that hibernating animals may experience cholestasis. Further work is needed to specifically establish if the gallbladder empties during the hibernation season.

Although the effects of hibernation were not examined, ground squirrels have been demonstrated to be an effective model for the investigation of gallstone production [22-25]. When fed high cholesterol diets or when treatment inhibited gallbladder motility in fed squirrels, these squirrels rapidly develop early clinical indications of gallstone formation such as cholesterol crystal formation. Gallstone formation is usually thought to occur when bile acids and lecithin concentrations are low as the cholesterol saturates bile acids and is thereby freed to participate in gallstone formation [13]. Both bile acids and lecithin were markedly reduced in the AB squirrels compared to both their winter and summer counterparts (Figs. 1A, 2A). Our frozen samples precluded assessment of microcrystal formation but we saw no indications of gallstones (personal observations). A mitigating factor for gallstone formation may be the anorexia experienced by AB squirrels; reduced gut activity may allow increased enterohepatic circulation of bile acids and greater binding of bile acids with free cholesterol and reduce the cholesterol saturation index [25]. High levels of protein are usually associated with increased nucleation times and incidence of cholesterol gall stone formation [26] but protein levels were lowest in the AB group (Fig. 2D).

In addition to the roles of bile acids in cholesterol metabolism and emulsification, there is an established role of bile acids as an endocrine signaler through several different motifs [27]. The primary regulatory role of circulating bile acids is in lipid metabolism. Bile acids may activate farnesoid × receptor α (FXRα) [28] and trigger regulation of cholesterol metabolism principally by modulation of hepatic 7α-hydroxylase expression [28,29]. It is tempting to speculate that the reduced bile acid levels found in the AB squirrels reflects an impaired cholesterol metabolism. However, levels of cholesterol were unchanged as a function of state (Fig. 1C) and further work on the dynamics of cholesterol formation and use during torpor are required before conclusions may be made.

Bilirubin concentrations were significantly higher in winter hibernators (IBA and T) as compared to summer squirrels and AB winter squirrels (Fig. 1B). Bilirubin is a product of erythrocyte and hemoglobin turnover [13] but no data are currently available for the fate of erythrocytes during hibernation. Although one might expect increased half-lives for these cells concordant with energetic demands during torpor, the markedly reduced body temperatures may cause significant cellular damage. A further examination of erythrocyte fate is warranted. Interestingly, higher bilirubin concentrations may confer protection against oxidative damage. Several studies have linked moderately elevated levels of blood bilirubin with greater ability to withstand oxidative stress through an anti-apoptotic role [30]. Furthermore, elevated blood bilirubin levels are associated with a decreased capacity for leukocytes to adhere to vasculature [31]. Leukocytes demonstrate reduced adhesion during hibernation and this diminished adhesion is thought to be involved with a natural ischemia tolerance exhibited by hibernators [32]. However, little information has been available as to a possible mechanism.

## Conclusion

This study was a first attempt to characterize the effects of hibernation on hepatobiliary function *per se*. Hibernators experience anorexia, metabolic fuel privation, and bouts of reduced body temperature. Yet despite these events, hibernator bile did not differ from summer squirrel bile in several key characteristics such as [bile acids], [cholesterol], [free fatty acids], [lecithin], and osmolality. One major distinction between summer and winter squirrels was that winter squirrels experience >5 fold increases in [bilirubin]. Such an increase may have significant physiological consequences that could aid in survivorship of torpor. Of note was that animals that failed to hibernate, despite being anorexic, were very similar to summer squirrels in all measured parameters except they had lower bile acid and lecithin concentrations. Our results highlight the need to further elucidate cholesterol metabolism during hibernation as well as understand the role of gallbladder contractility in determining bile constituents.

## Methods

Adult golden-mantled ground squirrels (*Spermophilus lateralis*) were captured during the summer from Southern Nevada and California. Some animals were trapped and killed immediately as a seasonal control (summer active, SA). The remaining squirrels were implanted in October with temperature sensitive radiotelemeters as described previously in order to allow for precise determination of torpor status [33]. Following recovery from surgery, implanted squirrels were housed in an environmental chamber at 4°C and allowed to hibernate. The body temperature of torpid squirrels was ~5°C. In some cases, torpor status was tracked through surface temperatures using an infrared thermometer. All animals were killed by CO_2 _asphyxiation except for the torpid animals. Torpid animals were killed by decapitation because of their low respiratory rates. The entire content of the gallbladder was collected to avoid stratification and the bile was snap frozen in liquid nitrogen and stored at -80°C until use. Bile was obtained from animals killed in the summer (SA), animals killed while torpid (T), and animals killed when euthermic between torpor bouts (interbout-aroused; IBA). An additional group of winter squirrels that failed to hibernate was included (deemed abnormal, AB). We note that these AB animals were implanted with telemeters at the same time (October), housed under the same conditions (4°C for more than two months), and sampled at the same time of year (~February) as the other winter squirrels. Animals received humane care according to the criteria outlined in the Guide for the Care and Use of Laboratory Animals (Institute of Laboratory Animal Resources, Washington, D.C., USA).

To assess for color variation, bile was photographed. Spectral analyses were also performed by diluting 1 μl of bile in 1 ml of water and scanning with a Shimadzu PharmaSpec Spectrophotometer (Shimadzu Scientific Instruments, Columbia, Maryland, USA) from 260 to 700 nm wavelengths at 0.5 nm resolution.

Bile acids were measured using a colorimetric assay. Four hundred μl of diluted bile was added to 1 ml of reaction mix consisting of 10 mM potassium phosphate buffer, pH 7.4, 50 U/l 3α-hydroxysteroid dehydrogenase, 0.1 mM nicotinamide adenine dinucleotide, 0.1 mM nitroblue tetrazolium, and 200 U/l diaphorase. Following incubation in the dark for 15 min at 37°C, sample absorbances were measured spectrophotometrically at 540 nm. Samples were compared against a standard curve using sodium taurocholate as a standard (r^2 ^of standard curve > 0.98).

Direct bilirubin concentrations were estimated colorimetrically through a commercial kit based on the production of azobilirubin and compared to a calibrator solution (Pointe Scientific, Canton, Michigan, USA). Duplicates of each bile sample were assayed and the mean was used for statistical analyses. Samples were in the manufacturer's indicated linear range of the assay.

Total cholesterol was estimated using a commercially available kit based on the production of the colorimetric product, quinoneimine (Pointe Scientific, Canton, Michigan, USA). Triplicates of each bile sample were assayed. Samples were compared against a standard curve using cholesterol as a standard (r^2 ^of standard curve > 0.98).

Free fatty acids were measured using the ADIFAB reagent (Molecular Probes, Eugene, Oregon, USA). ADIFAB was diluted in 50 mM tris-HCl, pH 8.0 and 1 mM EGTA to a stock solution of 13 μM. Just prior to use, the 13 μM stock solution was diluted to a 0.2 μM working solution with 10 mM potassium phosphate, pH 7.4. Two μl of bile or standard was added to 200 μl of ADIFAB working solution. Following 15 min incubation in the dark, fluorescence was measured at excitation of 392 nm and emission of 432 nm. Samples were compared to a standard curve constructed using equal parts of palmitic acid, stearic acid, oleic acid, linoleic acid, and linolenic acid dissolved in DMSO (r^2 ^of standard curve > 0.98). DMSO did not react with ADIFAB based on preliminary experiments (data not shown).

Lecithin/phosphatidylcholine was measured using a commercially available Phospholipids C kit (Wako Chemicals, Richmond, VA). The assay is based on the enzymatic cleavage of phospholipids to liberate choline which is oxidized in the presence of choline oxidase. The oxidation of choline liberates H_2_O_2 _which is detected using 4-aminoantipyrine. Triplicates of each bile sample were assayed. Values were compared to a standard curve using phosphatidylcholine (r^2 ^of standard curve > 0.99).

Bile pH was measured at 37°C using a calibrated Ultra M microelectrode (Lazar Research Laboratories, Los Angeles, California, USA). Osmolality was measured using a Vapro vapor pressure osmometer (Wescor, Logan, Utah, USA). One μl of bile was diluted with 9 μl of 150 mM NaCl and osmolality was measured. Values were then corrected by subtracting out the osmotic contribution of the 150 mM NaCl. This procedure allowed for use of the most sensitive range of the instrument.

Total protein was estimated through a modified Lowry protein assay [34]. Triplicates of each bile sample were assayed and compared against a standard curve using bovine serum albumin (r^2 ^of standard curve > 0.99).

## Competing interests

The authors declare that they have no competing interests.

## Authors' contributions

JAB and FvB participated equally in the assays. FvB was responsible for preparation of the manuscript. All authors read and approved the final manuscript.
